# Effects of Pomelo Peel-Derived Dietary Fibers on Simulated Intestinal Digestion and Fermentation of Fish Balls *In Vitro*

**DOI:** 10.3390/foods14101818

**Published:** 2025-05-20

**Authors:** Mingjing Zheng, Yiman Wei, Jinling Hong, Zhipeng Li, Yanbing Zhu, Tao Hong, Zedong Jiang, Hui Ni

**Affiliations:** 1 College of Ocean Food and Biological Engineering, Jimei University, Xiamen 361021, China; zmjfst@163.com (M.Z.); imane0902@163.com (Y.W.); 15006033804@163.com (J.H.); lzp2019@jmu.edu.cn (Z.L.); yanbingzhu@jmu.edu.cn (Y.Z.); zdjiang@jmu.edu.cn (Z.J.); nihui@jmu.edu.cn (H.N.); 2 Fujian Provincial Key Laboratory of Food Microbiology and Enzyme Engineering, Xiamen 361021, China; 3 School of Marine Biology, Xiamen Ocean Vocational College, Xiamen 361021, China

**Keywords:** pomelo peel dietary fiber, surimi product, simulated digestion *in vitro*, fermentation, gut microbiota

## Abstract

The effects of pomelo peel-derived dietary fibers (total dietary fiber, cellulose, and microcrystalline cellulose) on *in vitro* simulated gastrointestinal digestion and fermentation characteristics of silver carp fish balls were systematically investigated. Our findings revealed that pomelo peel dietary fibers significantly enhanced protein digestibility (highest increased by 18.58%), free amino acid content (most elevated by 13.27%), and slow digestion starch content (highest increased by 64.97%) in fish balls, suggesting an improved nutritional quality of fish balls. Moreover, pomelo peel-derived dietary fibers increased the content of short-chain fatty acids in the digestive fish balls at the late stage of fermentation (48 h) and caused changes in gut microbiota with reducing the ratio of *Firmicutes* to *Bacteroidetes* (F/B), the abundance of *Escherichia-Shigella* and *Streptococcus*, and increasing the levels of probiotics *Bacteroides* and *Phascolarctobacterium*. These suggested that pomelo peel-derived dietary fibers could promote the digestive characteristics of fish balls, effectively exerting prebiotic effects by regulating gut microbiota. The results could provide a scientific basis for the enhanced modification of intestinal digestion and fermentation of fish balls with dietary fibers.

## 1. Introduction

Pomelo (*Citrus maxima* (Burm.) Merri) is recognized as the largest citrus fruit, predominantly cultivated in subtropical and tropical regions [1,2]. China is rich in pomelo resources, with Fujian, Guangdong, and Guangxi being the primary producing areas. The outer peel of pomelo is relatively thick and light yellow or orange, accounting for approximately 40% to 50% of the fruit’s total weight [1]. In addition, pomelo peel contains 61.79% ± 0.87% (dry base) total dietary fiber [3], which is significantly higher than that in pulp [4]. The peel primarily consists of insoluble dietary fibers such as cellulose and hemicellulose (approximately 49.12% ± 1.19%) and soluble dietary fibers like pectin (around 14.07% ± 0.09%), making it an excellent source of dietary fiber [1,3,5]. Studies have shown that the dietary fiber of pomelo peel exhibits good physical and chemical properties, such as high water-holding capacity and expansibility, along with the functional benefits, e.g., lipid-lowering, blood sugar reduction, cholesterol reduction, gut microbiota improvement, antioxidant, and antibacterial effects [6,7,8,9]. Qi et al. have found that the total dietary fiber (TDF) content of pomelo fiber is higher than that of cereal fiber, and the properties such as water-holding capacity and viscosity are better than those of commonly used cereal and oat fibers [9]. The incorporation of 10% pomelo peel and soybean meal co-fermented protein was found to effectively remodel the intestinal microbiota, conducive to enhancing the immunity, hepatic, and intestinal health of largemouth bass [8]. Liu et al. [10] found that adding 3–9% rice bran insoluble dietary fiber could significantly delay the hydrolysis rate of rice flour starch. Dietary fiber can achieve an anti-obesity effect by reducing the ratio of *Firmicutes* to *Bacteroidetes* [11]. Fang et al. [12] showed that citrus dietary fiber could induce bladder cancer cell cycle arrest and cell apoptosis, thus significantly inhibiting tumor growth. It can be seen that adding pomelo peel-derived dietary fibers can not only change the quality of food but also improve the nutritional value of food.

Fish balls are a typical surimi product with high protein and low fat, and they are easy to eat. They are made of raw or frozen surimi, salt, starch, and other additives, followed by grinding, mixing, and heating to form the gel food [13]. Dietary fiber has been widely used to improve the texture, water-holding capacity, and nutritional characteristics of fish balls. For example, incorporating the fermented dietary fiber derived from bamboo shoots into the base material of fish balls can significantly reduce fat absorption, leading to the development of low-fat fried fish ball products [14]. Red algae powder with high dietary fiber can promote the formation of a dense gel structure of fish balls to increase its water-holding capacity, along with hypolipidemic activity for enhanced fish balls [15]. However, there are few reports studying the use of pomelo peel-derived dietary fibers as food ingredients, and the effects of different pomelo peel-derived dietary fibers on the digestion, absorption, and intestinal fermentation of fish balls still remain unknown.

The main components of dietary fiber from pomelo include cellulose, hemicellulose, lignin, pectin, etc. Dietary cellulose and microcrystalline cellulose from pomelo peel all belong to insoluble dietary fiber, where microcrystalline cellulose is a kind of dietary fiber with a higher crystallinity. Studies have shown that microcrystalline cellulose exhibits better thermal stability and water/oil holding ability than cellulose, and it also shows great prospects in the application of surimi products [2]. Therefore, three pomelo peel-derived dietary fibers including total dietary fibers, cellulose, and microcrystalline cellulose were prepared in this study; the effect of different pomelo peel-derived dietary fibers on digestive characteristics and the intestinal fermentation of fish balls were explored *in vitro*. Through the changes in protein and starch digestion, gut microbiota structure, and short-chain fatty acid (SCFA) content, the effects of different pomelo peel-derived dietary fibers on the potential intestinal health benefits of fish balls were elucidated. The results can lay a theoretical foundation for the regulation of the nutritional function of surimi products by dietary fiber and expand application pathways of pomelo peel-derived dietary fibers.

## 2. Materials and Methods

### 2.1. Materials

Pomelo peel was purchased from Fujian Pomelo town food Co., Ltd. (Zhangzhou, China). Fresh water grade AA surimi was purchased from Yasui Food Group Co., Ltd. (Xiamen, China). Composite phosphate (a mixture of sodium hexametaphosphate, sodium pyrophosphate, and sodium tripolyphosphate with a mass ratio of 1:1:1, all food grade) was bought from Hubei Xingfa Chemical Group Co., Ltd. (Yichang, China). Potato starch, salt, and TG enzyme were all commercially available. The other chemical reagents used were of analytical purity and purchased from Yuanye, Lablead, Sinopharm Group, Xilong Chemical, Aladdin, Solarbio and Macklin (Shanghai, Beijing, Beijing, Guangzhou, Shanghai, Beijing, Shanghai, China).

### 2.2. Preparation of Different Pomelo Peel-Derived Dietary Fiber

#### 2.2.1. Preparation of Total Dietary Fiber of Pomelo Peel

Pomelo peel was washed and then treated three times at 50 °C with a 60% ethanol solution at a ratio of 1:10 (*w*:*v*). The total dietary fiber of pomelo peel (named as PP; total dietary fiber content was determined to be around 85.90%) was obtained by drying, crushing, and passing through a 100-mesh screen.

#### 2.2.2. Preparation of Pomelo Peel-Derived Cellulose

The cellulose derived from pomelo peel was extracted by using the ultrasonic-assisted alkaline hydrogen peroxide method [16]. The pomelo peel was sliced, dried in the oven at 65 °C for 2 h, crushed, and screened with an 80-mesh screen. Then, 2.00 g pomelo peel powder was weighed, 0.02 g ethylenediaminetetraacetic acid (EDTA) and 0.01 g magnesium sulfate were added, the raw material 1:25 (g:mL) was added to the mixed solution of 0.90% H_2_O_2_ and 9% NaOH, and extraction occurred for 40 min under 320 W ultrasonic power and 78 °C water bath temperature. The extracted liquid was filtered (300-mesh filter cloth), washed, freeze-dried, and then crushed to obtain cellulose (labeled as PP-C).

#### 2.2.3. Preparation of Pomelo Peel-Derived Microcrystalline Cellulose

The pomelo peel-derived cellulose was mixed with a 7.80% hydrochloric acid aqueous solution at a solid–liquid ratio of 1:25 (g:mL) and extracted in an 82 °C water bath for 65 min. The solution was cooled for 10 min, filtered by a 300-mesh filter cloth, washed with distilled water, freeze-dried, and crushed to obtain microcrystalline cellulose (labeled as PP-MCC).

### 2.3. Preparation of Fish Balls with Pomelo Peel-Derived Dietary Fiber

Frozen silver carp surimi was thawed at 4 °C for 12 h and cut into small pieces, Then, 350 g of surimi was weighed and placed in a blender, chopped, and mixed for 2–4 min. A total of 10 g of salt was added, and it was ground for 7 min before 50 g of cassava starch, 1 g complex phosphate, 0.5 g TG enzyme, and 90 g ice water were added. The mixture was stirred for 6 min until the slurry was evenly mixed. Each 1%, 2%, and 3% w/w pomelo peel-derived dietary fiber (PP, PP-C, PP-MCC) was added to prepare different pomelo peel-derived fish balls; meanwhile, the one without pomelo peel-derived dietary fiber was labeled as the blank group. The evenly mixed slurry was squeezed into small balls, treated in a two-stage water heating gelation (30 min at 45 °C; 10 min at 90 °C), and then quickly put into ice water to cool for 20 min. The cooled fish balls were packaged and stored at −18 °C for further *in vitro* digestion and fermentation experiments.

### 2.4. Simulated Gastrointestinal Digestion In Vitro

The simulated oral, gastric, and intestinal digestion *in vitro* was conducted according to the methods of Brodkorb et al. [17] and Zheng et al. [18], in which saliva, gastric fluid, and intestinal fluid were prepared according to Minekus et al. [19].

The 5 g ground fish balls were mixed with 5 mL simulated saliva (pH 7.0, containing 75 U/mL α-amylase and 0.75 mM CaCl_2_) and shaken at 37 °C and 180 rpm for 2 min to simulate the process of chewing food in the human mouth. The sample was replaced by distilled water to prepare the control group.

After oral chewing, 10 mL oral chyme was mixed with 10 mL simulated gastric fluid (pH 3.0). The final pepsin activity was 2000 U/mL and CaCl_2_ concentration was 0.075 mM. The mixture was shaken at 37 °C and 180 rpm for 180 min to simulate human gastric digestion, and the digestive products were taken at 0, 30, 60, 90, 120, and 180 min, respectively.

A total of 20 mL gastric chyme was mixed with 20 mL simulated intestinal fluid (pH 7.0). The final activity of pancreatic α-amylase was 200 U/mL, the activity of α-glucosidase was 150 U/mL, and the concentrations of CaCl_2_ and bile acid were 0.3 mM and 10 mM, respectively. The solution was placed at 37 °C with shaking at 180 rpm for 180 min to simulate human intestinal digestion, and the digestive products were taken at 20, 40, 60, 90, 120, and 180 min, respectively.

These digestive products should be treated with enzyme elimination in a boiling water bath at 100 °C for 10 min and then subjected to the subsequent colon fermentation experiment [20].

### 2.5. Analysis of Gastric Digestive Products

#### 2.5.1. Determination of Protein Digestibility

The protein digestibility experiment was carried out according to the method of Cao et al. [21] with some modifications. The above gastric digestive products collected at different times were centrifuged at 4 °C and 13,201× *g* for 30 min. The protein content of the supernatant was determined by a Bicinchoninic Acid Assay (BCA). Protein digestibility *in vitro* was calculated as follows:

(1) $$\mathrm{Ptotein}\;\mathrm{digestibility}\;\mathrm{in}\;\mathrm{vitro}\;(\%)=\frac{M_{1}-M_{2}}{M_{0}}\times 100\%$$

where M_0_ is the total protein content (g) before fish ball digestion; M_1_ is the protein content (g) of the supernatant of the digestive products; and M_2_ is the protein content (g) of the digestive supernatant of the control group without fish balls.

#### 2.5.2. Analysis of Free Amino Acids in Gastric Digestive Products

The 22 kinds of amino acid standard substances were prepared with methanol or water and diluted with 10% formic acid methanol–water (1:1, *v*/*v*) to create an amino acid working standard solution. The appropriate amount of the isotope standard (Trp-d3) was weighed and prepared with 10% methanol to produce a mixed amino acid standard substance with a concentration of 1000 ng/mL. Sample pretreatment was modified according to the methods of Virág et al. [22] and Fuertig et al. [23]. A total of 400 μL 10% formic acid methanol–water was added into the supernatant of gastric digestive products, swirled for 30 s, and then centrifuged at 4 °C and 13,780× *g* for 5 min. The 20 μL of supernatant was added to 180 μL 10% formic acid methanol–water, swirled for 30 s, and mixed with 100 μL of the diluted sample with an isotope internal standard solution (100 ppb). The above mixed solution was passed through a 0.22 μm filter membrane for subsequent UPLC-MS detection.

Chromatographic condition: The chromatography was performed on an ACQUITY UPLC^®^ BEH C18 column (2.1 × 100 mm, 1.7 mm) with a sample volume of 5 μL. The column temperature was 40 °C. The mobile phase A was a 10% methanol–water solution (containing 0.1% formic acid) and B was a 50% methanol–water solution (containing 0.1% formic acid). The gradient elution procedure is as follows: 0–6.5 min, 10–30% B; 6.5–7.0 min, 30–100% B; 7–14 min, 100% B; and 14.0–17.5 min, 100–10% B. Flow rate was as follows: 0–8.0 min, 0.3 mL/min; 8.0–17.5 min, 0.4 mL/min [24,25].

Mass spectrum conditions: An electrospray ionization (ESI) source with positive ion ionization mode was used. The ion source temperature was 500 °C, the ion source voltage was 5500 V, the collision gas was 41.4 kPa, the gas curtain gas was 206.8 kPa, and the atomization gas and the auxiliary gas were 344.7 kPa. Multiple response monitoring was used for scanning [24,25].

### 2.6. Analysis of Intestinal Digestive Products

#### 2.6.1. Determination of Starch Digestibility

The determination of the starch digestible properties of fish balls was performed according to the method of Englyst [26]. The intestinal digestive products collected at different time periods (0, 20, 40, 60, 90, 120, and 180 min) were centrifuged at 4 °C and 13,201× *g* for 10 min, and then the glucose content in the supernatant was determined by the 3,5-dinitrosalicylic acid (DNS) method. The hydrolysis rate of starch was calculated by the following formula:

(2) $$\mathrm{Starch}\;\mathrm{hydrolysis}\;\mathrm{rate}\;(\%)=\frac{m_{GT}\times 0.9}{m_{TS}}\times 100\%$$

where m_GT_ is the glucose mass (mg) in digestive products at different time periods; 0.9 is the conversion coefficient of glucose to starch; and m_TS_ is the total starch mass (mg) in the sample.

#### 2.6.2. Determination of Relative Contents of Starch Components

Starch in food can be divided into fast digestible starch (RDS), slow digestible starch (SDS), and resistant starch (RS). RDS refers to starch digested within 20 min, which will increase the blood glucose response value rapidly. SDS refers to the starch digested within 20 to 120 min, which can better maintain the balance of the human blood glucose level. RS refers to the starch digested over 120 min and does not lead to a rapid rise in blood sugar [27]. Their relative content can be calculated according to the following formula [28]:

(3) $$\mathrm{Relative}\;\mathrm{content}\;\mathrm{of}\;\mathrm{RDS}\;(\%)=\frac{(m_{20}-m_{0})\times 0.9}{m_{TS}}\times 100\%$$

(4) $$\mathrm{Relative}\;\mathrm{content}\;\mathrm{of}\;\mathrm{SDS}\;(\%)=\frac{(m_{120}-m_{20})\times 0.9}{m_{TS}}\times 100\%$$

(5) $$\mathrm{Relative}\;\mathrm{content}\;\mathrm{of}\;\mathrm{RS}\;(\%)=\frac{TS-(RDS+SDS)}{m_{TS}}\times 100\%$$

where m_0_ is the free glucose mass (mg) in starch without hydrolysis; m_20_ is the glucose mass (mg) produced after hydrolysis of the sample for 20 min; m_120_ is the mass of glucose (mg) produced after hydrolysis of the sample for 120 min; 0.9 is the coefficient of converting glucose into starch; and m_TS_ is the total starch mass (mg) in the sample.

### 2.7. Simulated Colonic Fermentation with Gut Microbiota In Vitro

The *in vitro* fermentation simulation with gut microbiota for the intestinal digestive products of fish balls with different pomelo peel-derived dietary fibers was performed referring to the method of López-Barrera et al. [29] with slight modifications. Fresh fecal samples were taken from 5 volunteers (3 men and 2 women) who had not been treated with antibiotics or probiotics for at least 3 months and had no chronic disease.

Fresh fecal samples were mixed and diluted with PBS to obtain a fecal suspension (10%, *w*/*v*), centrifuged at a rate of 32× *g* for 10 min, and then the upper bacterial suspension was taken. The bacterial suspension was stored in 30% glycerol at −80 °C for subsequent experiments.

Preparation of basic nutrient medium (BNM): 2.0 g peptone, 0.1 g NaCl, 0.04 g K_2_HPO_4_, 0.04 g KH_2_PO_4_, 0.01 g CaCl_2_·6H_2_O, 0.01 g MgSO_4_ 7H_2_O, 0.2 g NaHCO_3_, 0.02 g heme chloride (soluble in 0.5 mL 1.0 M NaOH), 0.5 g L-cysteine hydrochloride anhydrous, 0.5 g III bile salt, 2.0 mL Tween-80, adjusted the pH to 7.4 ± 0.2, fixed volume to 1 L, and autoclaved at 121 °C for 20 min.

The bacterial suspension was inoculated into the brain heart infusion medium at 1% for activation and expansion, and the pH was adjusted to 7.4. The digested freeze-dried sample (1%, *w*/*v*) was added to BNM, and the BNM without the sample was used as the control group. The bacterial solution was mixed with the above BNM at a ratio of 1:1 and cultured in an anaerobic environment at 37 °C. With fermentation at 0, 12, 24, and 48 h, five parallel samples were taken, quenched with liquid nitrogen for 30 min, and then transferred for storage at −80 °C.

#### 2.7.1. Determination of pH of Fermentation Products

The fermentation solution was mixed evenly, and the pH was determined.

#### 2.7.2. Determination of Short-Chain Fatty Acids in Fermentation Products

A standard stock solution (100 mM) of acetic acid, propionic acid, butyric acid, and valeric acid was prepared. These standard solutions of 100, 50, 25, 10, 5, and 2.5 mM were prepared with ultra-pure water to draw the standard curves.

Short-chain fatty acids were extracted from fermentation products by ether extraction. The fermentation products were centrifuged at 4 °C and 13,201× *g* for 30 min, and the supernatant was collected. A fermentation supernatant of 1 mL was thoroughly mixed with 100 μL concentrated hydrochloric acid, 3 mL ether was slowly added, extraction was performed for 20 min, and then the mixture was centrifuged at 1548× *g* for 10 min to obtain the upper organic phase. The organic phase was mixed with 500 μL 1 M NaOH for 20 min and centrifuged at 1548× *g* for 10 min. The aqueous phase was extracted, and 100 μL concentrated hydrochloric acid was added, mixed, filtered by a 0.22 μm filter membrane, and analyzed by HPLC (LC-20AT, Shimadzu, Kyoto, Japan). The chromatography was performed on column C18 (4.6 mm × 150 mm), with a phosphoric acid buffer solution (pH 2.86) as mobile phase A and acetonitrile as mobile phase B, where mobile phase A/B (4:1), flow rate was 0.8 mL/min, the detection wavelength was 210 nm, and the sample size was 20 μL.

#### 2.7.3. Extraction of DNA and Sequencing of 16S rRNA

The DNA of each fermentation group was extracted by using the DNA extraction kit, and the purity and concentration of DNA was measured using Thermo NanoDrop One (ND-ONE-W, TMO, Wilmington, DE, USA). Using genomic DNA as a template, PCR amplification of V3-V4 of the 16S rRNA gene was performed using primers [30]. After the fragment length and concentration of PCR products were detected by 1% agarose gel electrophoresis, PCR mixed products were recovered using the E.Z.N.A.^®^ Gel Extraction Kit (Omega, Norcross, CA, USA) gel recovery kit, and target DNA fragments were recovered by TE buffer elution. The sequencing library was generated using the NEBNext^®^ Ultra^TM^ II DNA Library Prep Kit for Illumina^®^ (New EnglandBiolabs, Ipswich, MA, USA) according to the standard procedure. The constructed amplicon library was PE250-sequenced using the Illumina Nova 6000 platform.

Microbial 16S rDNA sequencing was commissioned by Guangdong Meg Gene Technology Co., Ltd.(Guangzhou, China), which was clustered on the Magichand cloud platform, and the sequences were clustered into OTUs (operational classification units), and then the abundance and diversity of gut microbiota were analyzed. The F/B ratio is then calculated as the ratio of the relative abundance of the phylum *Firmicutes* to that of the phylum *Bacteroidetes*.

### 2.8. Statistical Analysis

The experimental results are expressed as mean ± standard deviation (SD). A data processing system was applied to conduct a one-way ANOVA, and LSD multiple comparison was used to compare the significance of data using *p* < 0.05.

## 3. Results and Discussion

### 3.1. Effects of Different Pomelo Peel-Derived Dietary Fibers on Simulated Gastric Digestive Characteristics of Fish Balls In Vitro

#### 3.1.1. Changes in Protein Digestibility of Fish Balls

**Figure 1:**
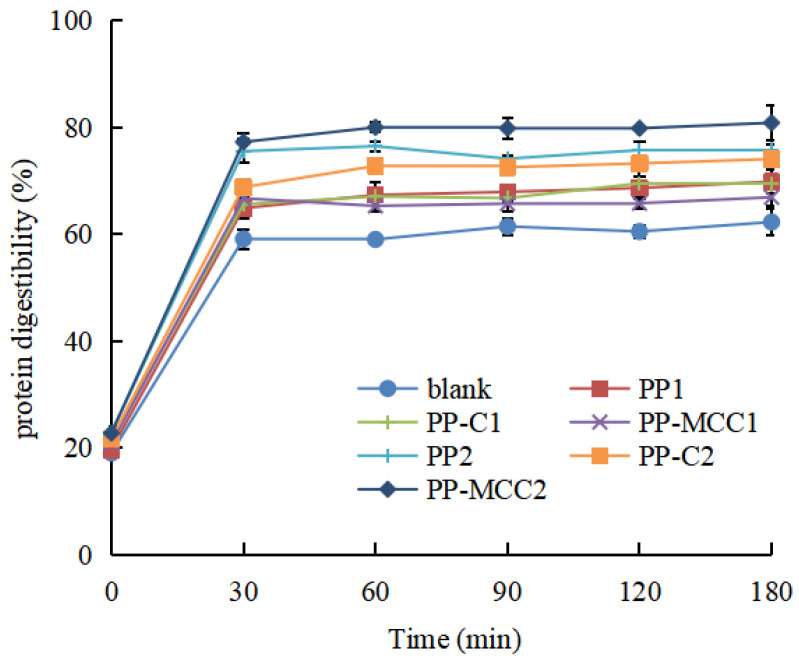
Effect of different pomelo peel-derived dietary fibers on protein digestibility in simulated gastric digestion of fish balls.

As shown in Figure 1, the protein digestibility of fish balls increased sharply when digested for 0–30 min, and then became slow after 30 min. The protein digestibility of fish balls supplemented with pomelo peel-derived dietary fiber were significantly higher than that of the blank group without pomelo peel-derived dietary fiber (*p* < 0.05), and the digestibility increased as the concentration of pomelo peel-derived dietary fiber increased.

Studies have shown that reducing the amount of undigested protein entering the colon can decrease protein degradation through gut microbiota fermentation, thus reducing the generation of harmful metabolites such as ammonia, amines, indole, and cresol, thereby promoting colonic health [31]. Therefore, we speculated that the addition of pomelo peel-derived dietary fiber can reduce the undigested protein entering the colon by promoting the protein digestibility in the stomach, thereby reducing harmful fermentation products of protein in the colon.

In this study, when the used dietary fiber content was 3%, the protein digestibility of fish balls with PP-MCC was the highest, increased by 18.59% as compared to the blank group without pomelo peel-derived dietary fiber, followed by 13.57% increased with pomelo peel total dietary fiber (PP) and 11.80% increased with pomelo peel cellulose (PP-C). This may be caused by the soluble dietary fiber in microcrystalline cellulose being almost removed. Soluble dietary fiber like κ-carrageenan can increase the viscosity of chyme and form a barrier film between enzymes and chyme, thus reducing the probability of contact between chyme and digestive enzymes and reducing digestibility [21,32]. With soluble dietary fiber removed in PP-MCC, the contact between the enzyme and the substrate was greater, thereby improving the protein digestibility of the fish balls.

#### 3.1.2. Changes in Free Amino Acids in Gastric Digestive Products

Protein hydrolysis by protease will produce small peptides and free amino acids; thus, the content of free amino acids can be used as an indirect indicator of protein digestion [33].

Table 1 shows the effects of different pomelo peel-derived dietary fibers on free amino acids in gastric digestive products of fish balls. Except 3% PP-MCC, the other pomelo peel-derived dietary fibers significantly increased the total amount of 22 kinds of free amino acids in the gastric digestive supernatant of fish balls (*p* < 0.05). As compared to the blank group without dietary fiber, the addition of 3% PP had the highest total amount of free amino acids, which were elevated by 13.27%, while the addition of 3% PP-C increased free amino acids by 9.85%. The total amount of free amino acids added with 3% PP-MCC did not significantly change (*p* ≥ 0.05) but was still 4.85% higher than that in the blank group. This was different from the above trend in protein digestibility, possibly because the proteins were hydrolyzed to produce a higher content of small peptides without free amino acids, which increased when PP-MCC was added.

In addition, the amino acids such as phenylalanine, leucine, lysine, and arginine are the main digestion sites of pepsin [34,35]. When 1% pomelo peel-derived dietary fibers were added, the contents of alanine, valine, threonine, and lysine in the gastric digestive supernatant were significantly higher than those in the blank group (*p* < 0.05), which may be due to the increased exposure of protein hydrophobic groups to improve the enzyme digestion efficiency of pepsin [33]. When 3% pomelo peel-derived dietary fibers was added, the contents of glycine, alanine, serine, proline, valine, threonine, isoleucine, aspartic acid, and glutamic acid were much higher than those in blank group (*p* < 0.05). The protein gel structure of the fish balls might be damaged; when more pomelo peel-derived dietary fiber was added, the pores became larger and looser and more disordered, which was conducive to protease entering the interior, thereby hydrolyzing the protein into more free amino acids [33].

It is worth mentioning that the total contents of nine essential amino acids, i.e., lysine, leucine, valine, isoleucine, threonine, phenylalanine, methionine, tryptophan, and histidine in fish balls supplemented with pomelo peel-derived dietary fibers were significantly higher than those in the blank group (*p* < 0.05). These nine amino acids are essential amino acids required by the human body, which are obtained from food and are usually utilized by cells at a rate commensurate with normal growth requirements and have become part of a healthy balanced diet [36]. The effects of 3% PP and PP-C on the essential amino acids of fish balls after simulated gastric digestion *in vitro* were better than that of 3% PP-MCC, which were consistent with the above results.

Overall, the results showed that pomelo peel-derived dietary fibers, especially total dietary fiber, can promote protein digestion and decomposition into more essential amino acids for human absorption and utilization, suggesting that fish balls with pomelo peel-derived dietary fibers have potential as a high-quality protein source.

### 3.2. Effects of Different Pomelo Peel-Derived Dietary Fibers on Simulated Intestinal Digestive Characteristics of Fish Balls In Vitro

**Figure 2:**
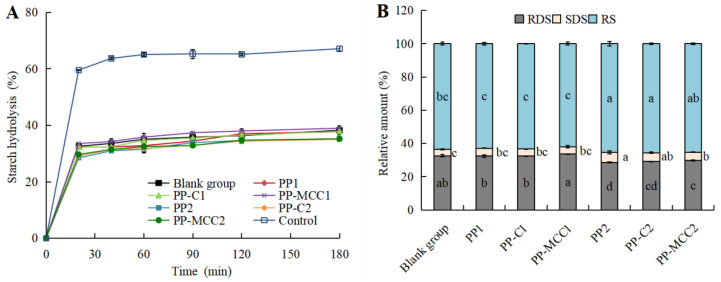
Starch hydrolysis curve (**A**) and composition and relative content (**B**) of fish balls with different pomelo peel-derived dietary fibers. Different lowercase letters in the figure indicate significant differences (*p* < 0.05).

The digestion rate of starch was the main factor affecting the release and absorption of glucose during the gastrointestinal digestion of food [37]. The effect of different pomelo peel-derived dietary fibers on simulated intestinal digestive characteristics of starch in fish balls was shown in Figure 2A. Taking white toast as the control food [38], the starch hydrolysis rate of fish balls increased sharply when digested initially in the small intestine for 20 min, increased slowly for 20–120 min, and tended to balance at 120–180 min. When digested for 180 min, the starch hydrolysis rate of white toast was 67.13% ± 0.87% and that of fish balls in the blank group was 38.23% ± 0.74%.

When compared with the blank group, the starch hydrolysis rate of fish balls was not affected with 1% pomelo peel-derived dietary fibers (*p* ≥ 0.05) but greatly decreased to around 35% through 3% PP, PP-C, and PP-MCC (*p* < 0.05). It may be because dietary fiber swells easily and has high viscosity, which can reduce the interaction between starch and amylase in fish balls and thus delay starch hydrolysis during *in vitro* intestinal digestion [39,40].

As can be seen from Figure 2B, the relative contents of RDS, SDS, and RS in the blank group were 32.58%, 3.74%, and 63.67%, respectively, and the addition of 1% pomelo peel-derived dietary fibers had no significant effect on the starch content of fish balls (*p* ≥ 0.05). The 3% pomelo peel-derived dietary fibers significantly decreased the relative content of RDS and increased the relative contents of SDS and RS (*p* < 0.05). Among pomelo peel-derived dietary fibers, the effect of PP was the most obvious, where the relative RDS content decreased by 12.74% and SDS increased by 64.97% with PP addition.

The results showed that a high concentration of dietary fiber in pomelo peel may inhibit the amylase hydrolysis to starch in fish balls [41], thereby reducing the content of RDS and increasing the contents of SDS and RS. Moreover, it has been reported that the intake of SDS and RS can delay the rise in postprandial blood sugar and reduce insulin levels [10,42]. Therefore, pomelo peel-derived dietary fibers can be used to develop functional fish balls with maintaining blood sugar balance, which has a potential consumer market.

### 3.3. Effects of Different Pomelo Peel-Derived Dietary Fibers on SCFAs in Fish Balls in In Vitro Simulated Fermentation

**Figure 3:**
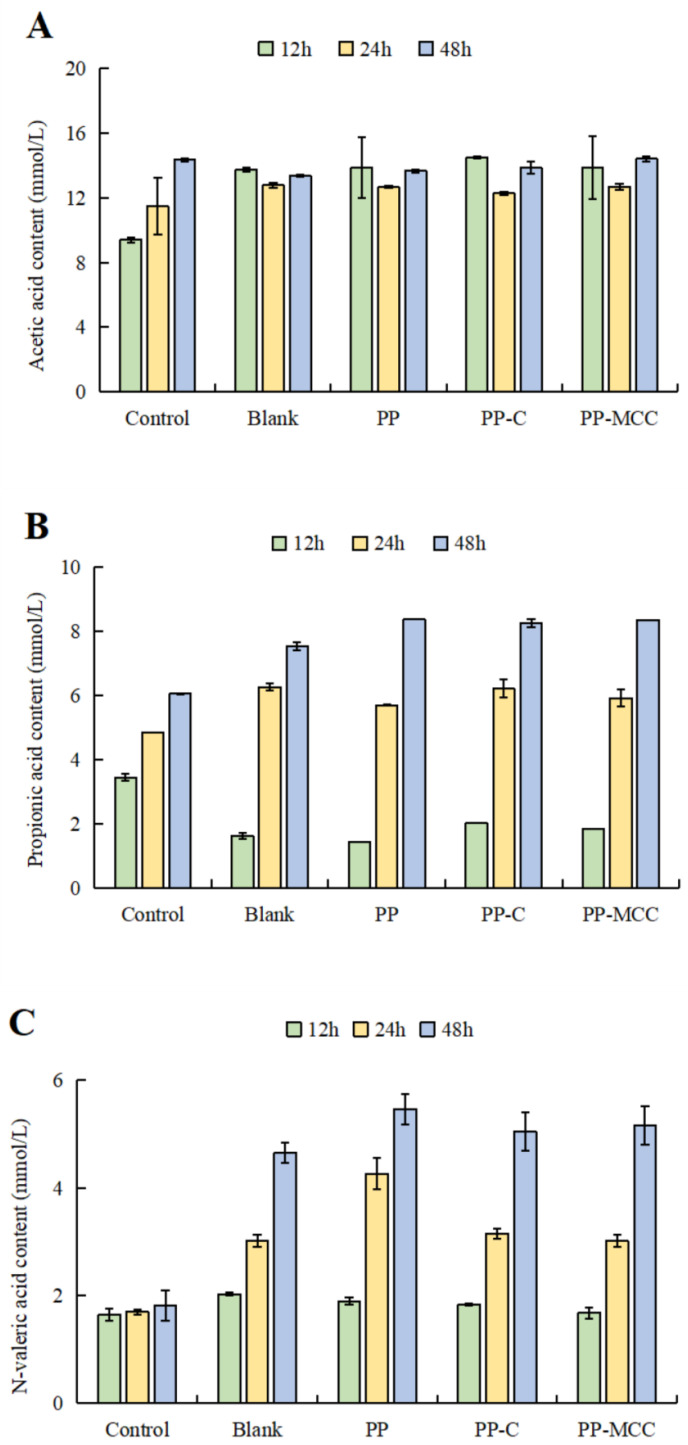
Changes in acetic acid (**A**), propionic acid (**B**), and N-valeric acid (**C**) during the fermentation of fish balls with different pomelo peel-derived dietary fibers.

SCFAs are derived from intestinal microbial metabolism, mainly including acetic acid, propionic acid, butyric acid, and valeric acid. The changes in SCFAs during the *in vitro* simulated fermentation of fish balls with gut microbiota affected by different pomelo peel-derived dietary fibers are shown in Figure 3. With fish ball fermentation, the main SCFAs were acetic acid, propionic acid, and N-valeric acid, and the content of acetic acid was the highest among those SCFAs. However, butyric acid was not detected in the fermentation solution, probably because the content was too low to detect.

As shown in Figure 3A, compared to the blank group, when fish balls containing pomelo peel-derived dietary fibers were fermented by the human gut microbiota, the acetic acid content was significantly increased at 12 h, slightly decreased at 24 h, and then increased at 48 h of fermentation.

The acetic acid content of fish balls with pomelo peel-derived dietary fibers was higher than that of the blank group. The changes in propionic acid content are shown in Figure 3B. Compared with the control group, the propionic acid content in blank fish ball fermentation was greatly increased at 24 h and 48 h (*p* < 0.05). Compared with the blank group, the propionic acid content in the fermentation of fish balls with pomelo peel-derived dietary fibers was significantly increased at 48 h (*p* < 0.05), and the content of fish balls containing PP was the highest, which was 38.25% higher than that in the blank group (*p* < 0.05).

Studies have reported that the content of acetic acid and propionic acid can be increased after the fermentation of undigested starch and dietary fiber [43,44]. The increased SDS and RS and pomelo peel-derived dietary fibers in digestive products of fish balls promote the production of more acetic acid and propionic acid in the late gut microbiota fermentation period (48 h). Similarly, the content of N-valeric acid in the fermented products of fish balls with PP at 48 h was higher than that in the blank group (Figure 3C). Acetic acid and propionic acid, as classical SCFAs, primarily participate in important physiological functions such as metabolism and immunity [45,46]. Valeric acid has been found to suppress autoimmunity by regulating lymphocyte metabolism and may also serve as a potential cancer therapeutic agent [47,48]. In particular, short-chain fatty acids have been found to be associated with obesity. For instance, weight loss caused by dietary restrictions is usually accompanied by a decrease in the concentration of SCFAs [49]. Acetate regulated the levels of DNA methylation at the host miR-378a promoter, thus preventing the development of obesity [50]. In addition, SCFAs also showed a positive effect in regulating vascular inflammation [51].

In conclusion, fish balls supplemented with pomelo peel-derived dietary fibers can generate more acetic acid, propionic acid, and valeric acid at 48 h of gut microbiota fermentation, especially the addition of total dietary fiber PP for increasing propionic acid and valeric acid, which has the potential to improve diet-induced obesity and inflammation and thus promote human intestinal health.

### 3.4. Effects of Different Pomelo Peel-Derived Dietary Fibers on Gut Microbiota Diversity in Fish Balls in In Vitro Simulated Fermentation

#### 3.4.1. OTU Level of Gut Microbiota

**Figure 4:**
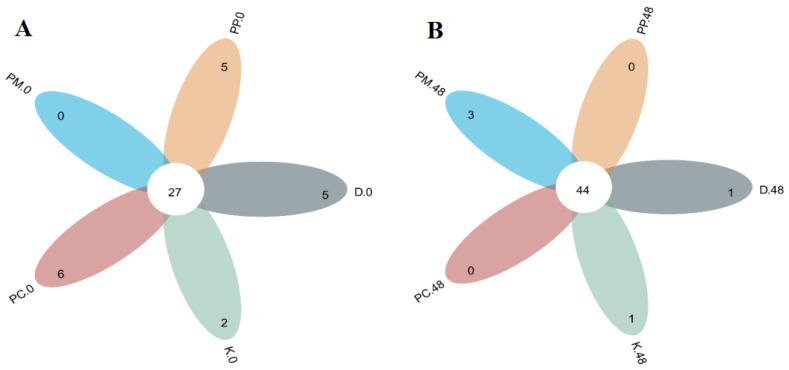
Venn diagram for OTUs of gut microbiota in fermentation products of fish balls with different pomelo peel-derived dietary fibers at 0 h (**A**) and 48 h (**B**).

The digestive products of fish balls with different pomelo peel-derived dietary fibers were used for the *in vitro* fermentation of human gut microbiota. The OTU levels of gut microbiota were analyzed using a Venn diagram, as shown in Figure 4, where different colors represent different samples, the overlapping area of the circle represents the common OTU, and the non-overlapping area represents the unique OTU. The total number of OTUs at fermentation for 0 and 48 h were 27 and 44, respectively, among which the total number at 0 h was the lowest and the total number at 48 h was the highest. With the extension of fermentation time, the total number of OTUs increased, indicating that the similarity of gut microbiota generated by fermentation increased and the unique bacteria of different samples decreased. The unique OTU numbers among the PP, PP-C, and PP-MCC groups were five, six, and zero, respectively, at 0 h. After 48 h of fermentation, the specific OTU number of the PP and PP-C groups was 0, while the unique OTU number in the PP-MCC group increased to three.

These results indicate that with the increase in fermentation time, the similarity of gut microbiota in the total dietary fiber group and cellulose group increased, while the microcrystalline cellulose group produced more specific bacteria.

In conclusion, the addition of dietary fiber in pomelo peel can help digestive products of fish balls regulate the structure of gut microbiota, which is closely related to its type.

(D.0, D.48 indicates that the control group was fermented at 0 h and 48 h; K.0, K.48 indicates that the blank group was fermented for 0 h and 48 h; PP.0, PP.48 indicates that the fish balls supplemented with the total dietary fiber of pomelo peel were fermented for 0 h and 48 h; PC.0, PC.48 indicates that the fish balls added with pomelo peel cellulose were fermented for 0 h and 48 h; PM.0, PM.48 indicates that the fish balls added with pomelo peel microcrystalline cellulose were fermented for 0 h and 48 h, the same as below.)

#### 3.4.2. α-Diversity of Gut Microbiota

**Figure 5:**
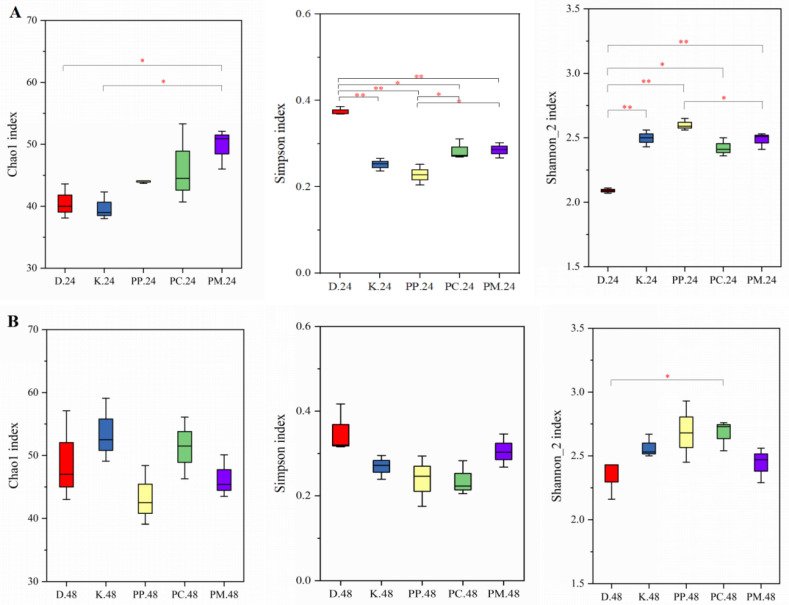
Changes in the structure of gut microbiota at α-diversity during the *in vitro* fermentation of fish balls with different pomelo peel-derived dietary fibers (**A**,**B**) represent fermentation for 24 and 48 h, respectively; * *p* < 0.05, ** *p* < 0.01).

Chao1, Simpson, and Shannon_2 indices were used to evaluate the α-diversity of gut microbiota during the fermentation of fish ball digestive products, and the results are shown in Figure 5. At 24 h of fermentation (Figure 5A), the Chao1 index of the PP-MCC group was significantly higher than that of the control group and blank group (*p* < 0.05), and the Simpson index of the PP group was the lowest, with a higher Shannon_2 index than that of the PP-MCC group and control group. The results suggested that the addition of PP-MCC can improve microbial richness in the fish ball fermentation solution at 24 h and the microbial uniformity is higher than that of the PP group, but the PP group has higher diversity, which may increase the number of rare and dominant OTUs in the microbiota [52]. However, after 48 h of fermentation (Figure 5B), the addition of dietary fiber of pomelo peel had no effect on the richness, uniformity, and diversity of gut microbiota in the fish ball fermentation solution (*p* > 0.05).

In conclusion, the addition of different types of pomelo peel dietary fiber in fish balls had effects on the α-diversity of gut microbiota at different periods of fermentation, but the effect was greater at 24 h of fermentation.

### 3.5. Effects of Different Pomelo Peel-Derived Dietary Fibers on Structure of Gut Microbiota During Fish Ball In Vitro Simulated Fermentation

**Figure 6:**
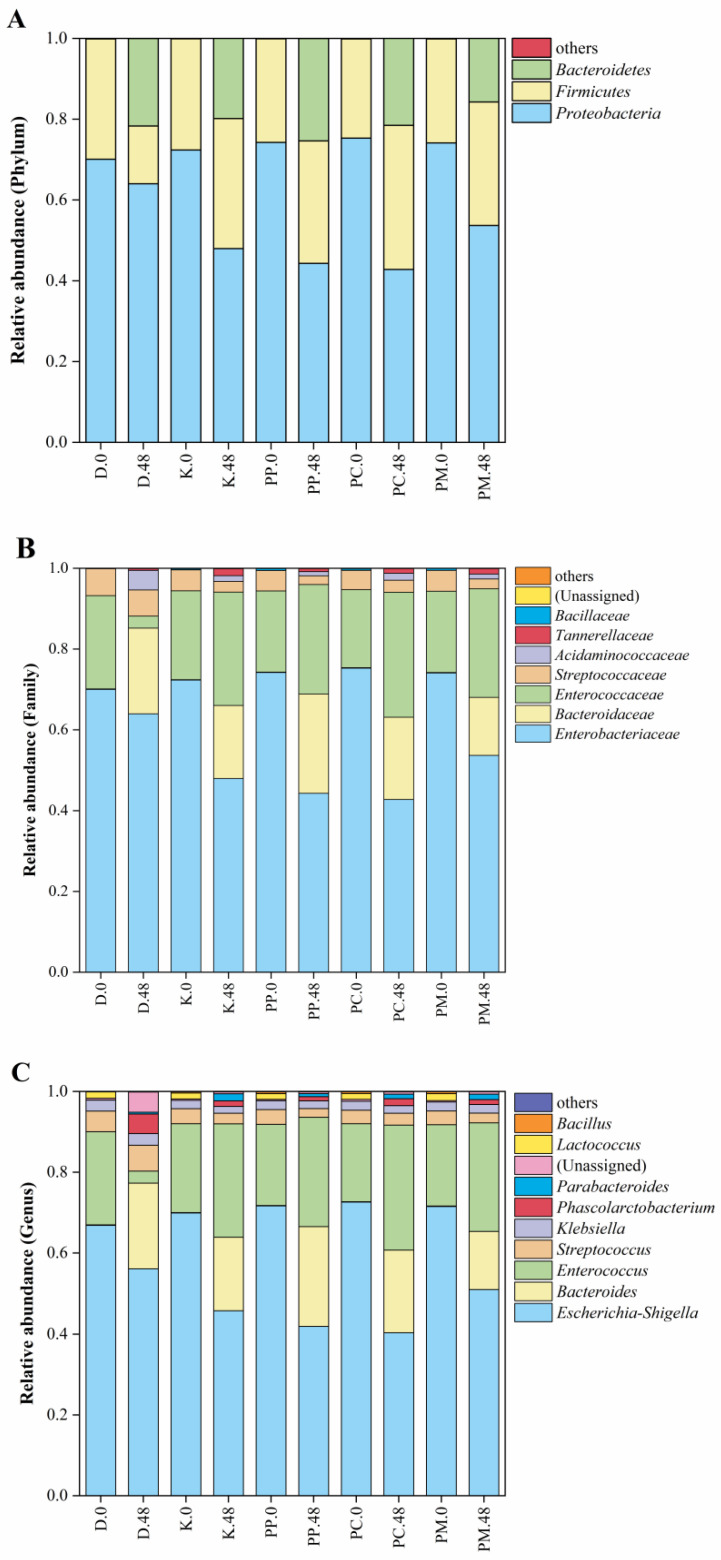
Changes in the structure of gut microbiota at phylum (**A**), family (**B**), and genus (**C**) levels during the *in vitro* fermentation of fish balls with different pomelo peel-derived dietary fibers.

Figure 6 shows the changes in gut microbiota at 48 h of fermentation of fish ball digestive products with different pomelo peel-derived dietary fibers. At the phylum level (Figure 6A), compared with the blank group, the fish balls supplemented with different pomelo peel-derived dietary fibers reduced the F/B ratio of gut microbiota after fermentation. Studies have found that *Bacteroidetes* can degrade polysaccharides and fibers that are not digested by the upper digestive tract [53]. Therefore, the above decreased F/B ratio may be due to the utilization of the starch and dietary fiber in the digestive products of fish balls by *Bacteroidetes*, thus increasing the abundance of *Bacteroidetes* and decreasing the abundance of *Firmicutes*.

Moreover, the fish balls supplemented with PP and PP-C can reduce the abundance of *Proteobacteria*, while the one with PP-MCC increased the abundance of *Proteobacteria*. Studies have shown that changes in the F/B value and *Proteobacteria* abundance in gut microbiota are negatively related to obesity [11,30]. This suggests that different pomelo peel-derived dietary fibers, especially PP and PC, can be explored to enhance the regulation of gut microbiota and anti-obesity for fish balls.

At the family level (Figure 6B), compared with the control group, the relative abundance of *Enterobacteriaceae* and *Streptococcaceae* was decreased by fish ball fermentation at 48 h. Compared with the blank group without pomelo peel-derived dietary fibers, the relative abundance of *Enterobacteriaceae* decreased and that of *Bacteroideaceae* increased in the fish balls with the PP and PP-C group at 48 h fermentation, while the effect of the PP-MCC group showed the opposite effect. Studies have found that *Enterobacteriaceae* was a conditional pathogen, which was a major cause of morbidity and mortality worldwide [54], while *Bacteroideaceae* was a beneficial bacterium for intestinal health [55]. *Bacteroideaceae* can produce acetic acid and propionic acid [56], which is consistent with the above short-chain fatty acid change results.

However, the propionic acid content of PP-MCC at this time was also higher than that of the blank group, which is inconsistent with the relatively abundant results of *Bacteroideaceae*, possibly because the other enhanced *Enterobacteriaceae* can also produce propionic acid [57]. It is inferred that pomelo peel-derived dietary fibers, especially cellulose and total dietary fiber, can positively regulate the gut microbiota of fish ball digestive products.

At the genus level (Figure 6C), the relative abundances of *Escherichia coli* and *Streptococcus* in the digestive products of fish balls without pomelo peel-derived dietary fibers after fermentation were lower, while the relative abundances of *Parabacteroides*, *Enterococcus*, and *Phascolarctobacterium* were increased and the relative abundances of *Bacteroides* in the PP and PP-C groups were significantly higher than those in the blank groups. It has been reported that *Bacteroides* can utilize a large number of food substrates or components, especially proteins and carbohydrates that have biofunctional metabolites [58].

According to the above experimental results, SDS and RS in the 3% PP and PP-C groups were higher than in the blank group and PP-MCC group. Therefore, more components were utilized by *Bacteroides* in the 3% PP and PP-C group than the blank group and PP-MCC group, thus increasing the relative abundance of *Bacteroides* in the late fermentation period. A high abundance of *Bacteroides* can promote the production of more SCFAs such as acetic acid and propionic acid. These SCFAs can improve obesity and maintain intestinal barrier function by regulating energy metabolism and reducing intestinal permeability. Moreover, the reduction in the relative abundance of harmful bacteria like *Enterobacteriaceae* results in a decrease in endotoxin release and a reduction in the inflammatory level, thus delaying the onset of inflammation [59]. It can be seen from this that pomelo peel-derived dietary fibers PP and PP-C achieve the purpose of maintaining good health by promoting the growth of beneficial bacteria and inhibiting the proliferation of harmful bacteria.

## 4. Conclusions

The effects of pomelo peel-derived dietary fibers on the simulated intestinal digestion and fermentation of fish balls *in vitro* was studied. The above results showed that different types of pomelo peel-derived dietary fibers can promote the digestion of proteins in fish balls, increasing the content of nine essential amino acids. Moreover, adding 3% pomelo peel-derived dietary fibers could inhibit the hydrolysis of starch *in vitro*, along with an increase in the contents of SDS and RS. The addition of pomelo peel-derived dietary fibers, especially PP, increased the contents of acetic acid, propionic acid, and n-valeric acid, especially propionic acid, at the late stage of fermentation (48 h). In addition, it was found that adding pomelo peel-derived dietary fibers PP and PP-C could increase the species richness of gut microbiota at the later stage of fermentation, in which the abundances of beneficial bacteria such as *Bacteroides*, *Phascolarctobacterium*, and *Parabacteroides* were increased, while the abundances of harmful bacteria such as *Escherichia-Shigella* and *Streptococcus* were decreased. It can be seen that fish balls with pomelo peel-derived dietary fibers PP and PP-C have a positive regulatory effect on gut microbiota and have potential intestinal health benefits. The results suggested that pomelo peel-derived dietary fibers can be explored as a raw material in surimi products. It can not only effectively enhance the nutritional value of surimi products and aid in the development of new functional fish ball products but also improve the utilization rate of pomelo by-products, reducing resource waste and increasing the added value of pomelos simultaneously.

## Figures and Tables

**Table 1:** Effect of different pomelo peel-derived dietary fibers on free amino acids in the supernatant of fish ball gastric digestion.

Content (μg/mL)		Blank Group	PP1	PP-C1	PP-MCC1	PP2	PP-C2	PP-MCC2
Special amino acids	GABA	0.76 ± 0.00 ^d^	0.86 ± 0.00 ^b^	0.80 ± 0.01 ^cd^	0.82 ± 0.01 ^bc^	0.94 ± 0.05 ^a^	0.81 ± 0.02 ^cd^	0.83 ± 0.01 ^bc^
	Orn	15.22 ± 0.36 ^c^	15.82 ± 0.38 ^bc^	16.11 ± 0.31 ^ab^	15.77 ± 0.10 ^bc^	16.65 ± 0.57 ^a^	15.83 ± 0.25 ^bc^	15.96 ± 0.34 ^abc^
	Hcy	0.22 ± 0.03 ^ab^	0.22 ± 0.01 ^ab^	0.18 ± 0.01 ^c^	0.18 ± 0.01 ^bc^	0.18 ± 0.03 ^c^	0.19 ± 0.00 ^bc^	0.24 ± 0.02 ^a^
Non-essential amino acids	Gly	43.93 ± 0.30 ^d^	48.24 ± 0.62 ^bc^	42.71 ± 0.62 ^d^	49.22 ± 0.19 ^ab^	51.01 ± 2.20 ^a^	48.37 ± 0.43 ^bc^	46.86 ± 1.09 ^c^
	Ala	62.27 ± 0.07 ^c^	66.52 ± 1.87 ^b^	67.21 ± 1.67 ^b^	68.15 ± 0.53 ^ab^	72.02 ± 3.71 ^a^	67.84 ± 1.72 ^b^	68.56 ± 0.19 ^ab^
	Ser	28.33 ± 0.53 ^c^	29.63 ± 0.76 ^bc^	30.05 ± 0.41 ^b^	30.14 ± 0.78 ^b^	31.99 ± 0.99 ^a^	30.72 ± 0.12 ^ab^	30.13 ± 0.38 ^b^
	Pro	15.01 ± 0.17 ^c^	15.79 ± 0.40 ^bc^	16.29 ± 0.21 ^ab^	16.43 ± 0.00 ^ab^	17.02 ± 0.86 ^a^	15.99 ± 0.07 ^b^	16.10 ± 0.15 ^b^
	Asn	42.35 ± 3.23 ^de^	53.33 ± 3.74 ^ab^	44.01 ± 1.01 ^cd^	56.22 ± 1.42 ^ab^	50.30 ± 3.82 ^bc^	58.27 ± 4.61 ^a^	36.18 ± 2.52 ^e^
	Asp	37.20 ± 0.04 ^c^	40.42 ± 0.93 ^b^	40.53 ± 1.60 ^b^	39.45 ± 0.40 ^bc^	45.31 ± 2.75 ^a^	40.67 ± 0.91 ^b^	40.18 ± 0.20 ^b^
	Gln	8.68 ± 0.03 ^a^	8.92 ± 0.07 ^a^	9.21 ± 0.00 ^a^	8.82 ± 0.34 ^a^	8.72 ± 0.50 ^a^	8.96 ± 0.23 ^a^	9.00 ± 0.04 ^a^
	Glu	46.95 ± 1.24 ^c^	51.54 ± 0.31 ^ab^	50.10 ± 1.59 ^bc^	51.83 ± 0.14 ^ab^	54.27 ± 2.86 ^a^	51.69 ± 1.77 ^ab^	51.88 ± 1.05 ^ab^
	Arg	19.83 ± 0.26 ^b^	20.65 ± 0.15 ^b^	20.94 ± 0.14 ^b^	20.24 ± 0.06 ^b^	22.54 ± 1.73 ^a^	20.64 ± 0.60 ^b^	20.29 ± 0.39 ^b^
	Tyr	47.96 ± 0.81 ^b^	47.63 ± 0.87 ^b^	49.01 ± 1.05 ^ab^	48.28 ± 0.61 ^ab^	52.03 ± 4.40 ^a^	47.74 ± 0.66 ^b^	46.74 ± 0.21 ^b^
Essential amino acid	Val	47.95 ± 0.97 ^c^	53.22 ± 1.13 ^b^	54.09 ± 0.60 ^b^	53.99 ± 0.13 ^b^	58.33 ± 1.87 ^a^	54.86 ± 0.77 ^b^	53.33 ± 2.49 ^b^
	Thr	27.80 ± 0.18 ^c^	30.79 ± 0.06 ^ab^	30.22 ± 0.01 ^b^	30.46 ± 0.22 ^b^	31.52 ± 0.73 ^a^	30.89 ± 0.77 ^ab^	30.37 ± 0.08 ^b^
	Ile	42.23 ± 0.10 ^c^	44.52 ± 0.67 ^bc^	45.29 ± 1.33 ^abc^	46.77 ± 0.02 ^ab^	48.29 ± 2.75 ^a^	47.05 ± 2.11 ^ab^	45.92 ± 0.36 ^ab^
	Leu	56.91 ± 0.56 ^b^	60.21 ± 1.22 ^ab^	59.98 ± 2.77 ^ab^	59.72 ± 0.46 ^ab^	62.26 ± 2.51 ^a^	58.89 ± 1.90 ^ab^	58.75 ± 0.74 ^ab^
	Lys	38.10 ± 0.09 ^b^	40.51 ± 1.15 ^a^	41.35 ± 0.81 ^a^	40.84 ± 0.05 ^a^	42.14 ± 1.66 ^a^	40.59 ± 0.63 ^a^	40.21 ± 1.34 ^ab^
	Met	34.42 ± 0.05 ^b^	35.94 ± 1.83 ^ab^	35.91 ± 0.84 ^ab^	35.97 ± 1.09 ^ab^	37.71 ± 1.48 ^a^	38.38 ± 0.00 ^a^	36.00 ± 1.51 ^ab^
	Phe	150.37 ± 0.19 ^d^	159.75 ± 2.59 ^abc^	161.19 ± 4.01 ^abc^	156.25 ± 1.19 ^cd^	166.67 ± 8.04 ^a^	165.79 ± 2.87 ^ab^	157.48 ± 4.05 ^bcd^
	Trp	20.54 ± 0.05 ^bc^	20.92 ± 0.37 ^abc^	20.85 ± 0.75 ^abc^	21.59 ± 0.27 ^ab^	21.90 ± 1.25 ^a^	20.80 ± 0.05 ^abc^	20.28 ± 0.20 ^c^
	His	7.81 ± 0.07 ^b^	8.26 ± 0.07 ^ab^	8.35 ± 0.14 ^a^	8.10 ± 0.21 ^ab^	8.51 ± 0.54 ^a^	8.22 ± 0.11 ^ab^	8.09 ± 0.13 ^ab^
Total essential amino acid		426.12 ± 0.06 ^c^	454.11 ± 8.83 ^b^	457.24 ± 9.26 ^ab^	453.70 ± 2.03 ^b^	477.33 ± 20.82 ^a^	465.47 ± 9.21 ^ab^	450.43 ± 10.02 ^b^
Total free amino acid		794.83 ± 4.52 ^c^	853.69 ± 17.54 ^b^	844.40 ± 15.78 ^b^	859.26 ± 5.54 ^ab^	900.31 ± 37.66 ^a^	873.18 ± 18.30 ^ab^	833.39 ± 16.12 ^bc^

Different lowercase letters in the same line indicate significant differences (*p* < 0.05). The blank is the fish ball without adding pomelo peel dietary fiber. PP1 and PP2 are fish balls supplemented with 1% and 3% total dietary fiber of pomelo peel. PP-C1 and PP-C2 are fish balls with 1% and 3% pomelo peel cellulose. PP-MCC1 and PP-MCC2 are fish balls with 1% and 3% pomelo peel microcrystalline cellulose.

## Data Availability

The original contributions presented in the study are included in the article; further inquiries can be directed to the corresponding author.

## Abbreviations

The following abbreviations are used in this manuscript:

PP	Fish balls supplemented with total dietary fiber of pomelo peel
PP-C	Fish balls with pomelo peel cellulose
PP-MCC	Fish balls with pomelo peel microcrystalline cellulose
